# Can ovarian double-stimulation in the same menstrual cycle improve IVF outcomes?

**DOI:** 10.5935/1518-0557.20170042

**Published:** 2017

**Authors:** Maria Cecília de Almeida Cardoso, Alessandra Evangelista, Cássio Sartório, George Vaz, Caio Luis Vieira Werneck, Fernando Marques Guimarães, Paulo Gallo de Sá, Maria Cecília Erthal

**Affiliations:** 1 Vida - Centro de Fertilidade, Rio de Janeiro, Brazil.; 2 Department of Gynecology of the State University of Rio de Janeiro (UERJ).

**Keywords:** oocytes, ovarian stimulation, *in vitro* fertilization, blastocyst

## Abstract

**Objective:**

To evaluate the double-stimulation protocol efficacy over conventional ovarian stimulation in recovering a more adequate number of oocytes and increase the number of embryos to be transferred or to be genetically analyzed.

**Methods:**

A retrospective and comparative study with 13 patients who underwent unsuccessful *in vitro* fertilization (IVF) cycles with a conventional antagonist ovarian stimulation protocol and repeat the attempt with a double stimulation protocol. The following variables were analyzed: number of oocytes collected, mature oocytes collected, fertilization rate, blastocyst rate, biopsied blastocyst rate and euploidy rate.

**Results:**

The double stimulation protocol had a significant higher number of oocytes collected (*p*=0.007) and mature oocytes to be injected (*p*=0.01). There was no statistically significant difference in fertilization (*p*=0.78) and blastocyst (*p*=0.59) rates.

**Conclusion:**

Double stimulation favors patients who are at risk of incurring several attempts of IVF to achieve pregnancy.

## INTRODUCTION

The increasing number of women who delay pregnancy and must undergo assisted reproductive technology (ART), poses the challenge of finding increasingly efficient ovarian stimulation protocols, since oocyte donation is not always accepted. It is known that 76% of the blastocysts produced from women older than 40 years are aneuploid (Harton *et al.*, 2013). In these women, the ovarian response is below ideal and worsens with the interval between treatments.

The number of oocytes used in *in vitro* fertilization (IVF) is directly related to the reproductive outcome. Patients with few oocytes are less likely to get pregnant and pose a great challenge for specialists (Polyzos & Devroey, 2011). Therefore, the shorter the time, the greater the number of eggs obtained and the higher the likelihood of reaching an embryo with potential for implantation and development of a full-term pregnancy. However, an aggressive stimulation, in addition to the risk of hyperstimulation, may recover lower quality oocytes due to the risk of premature luteinization.

Baerwald *et al*. (2003) demonstrated that, during the luteal phase, remaining small antral follicles could be in the early stages of follicular development, suggesting that the ovary could have been continuously stimulated during the menstrual cycle. That possibility has proved to be especially useful in fertility preservation for patients in a hurry to initiate cancer treatment. Aware of that, and excited about a patient's outcome who accidentally had a luteal-phase ovarian stimulation (LPS), Kuang *et al.* (2014) studied the efficacy of initiating ovarian stimulation in the luteal phase, so they could extend the concept to a routine IVF setting that could be used independently of menstruation. The study demonstrated that luteal phase stimulation (LPS) is appropriate in producing competent oocytes, and consequently, embryos with good pregnancy outcomes, with the advantage of eradicating the ovarian hyperstimulation syndrome (OHSS) or premature luteinization. Other authors corroborated the LPS protocol feasibility (Lin *et al*., 2016; Wang *et al*., 2016; Wei *et al*., 2016), and the same group, later, described the safety for the offspring originated from that protocol (Chen *et al*., 2015).

The theory that folliculogenesis occurs in a wave-like fashion and that there are multiple follicular recruitment waves in the same menstrual cycle (Baerwald *et al*., 2012), coupled to the LPS protocol success, was an inspiration for another stimulation, proposed to benefit patients with poor ovarian response (POR). Also in 2014, Kuang proposed a new protocol for ovarian stimulation, called Shangai Protocol, because it was presented during the BCGIP-COGI in Xangai. The strategy is to use luteal phase ovarian stimulation following oocyte retrieval, in the same cycle when follicular phase ovarian stimulation had already been carried out. With the main purpose of retrieving more oocytes in a short period of time, they used letrozole or clomiphene citrate plus hMG, ovarian LH surge suppression with GnRH-antagonist and its triggering with GnRH-agonist, associated with total embryo vitrification. The one thing they did different with this protocol was the sequential stimulation including the luteal phase. As the established protocols are unable to make the poor responder to have a normal response, this approach, called double ovarian stimulation (Kuang *et al*., 2014), aims to obtain the highest number of oocytes in the shortest time, thus avoiding the waste of time, crucial in this type of patient, in repeated attempts (Zhang, 2015).

Ubaldi *et al*. (2016) proposed the double stimulation protocol, which they called DuoStim, for patients with reduced ovarian reserve, taking into account the "time as an important factor for all patients, but it is crucial for those with have a foreseeable rapid loss/decrease of fertility". Different from the Xangai protocol, which used letrozole or clomiphene citrate plus hMG, they used recombinant gonadotrophins (FSH and LH), and after 5 days of the oocyte retrieval, a luteal phase stimulation was started like the previous stimulation. The aim of the study was to exploit the ovarian reserve to increase the offer of euploid embryos to transfer per intention to treat. They could increase the rate of euploid embryos from 41.9% (from oocytes exclusively obtained from follicular phase stimulation) to 69.8% considering cumulate oocytes from both follicular and luteal phase stimulation.

We started to offer the double stimulation protocol, in May 2016 for fertility preservation (oocyte cryopreservation) and IVF to patients with POR, fertilization failure in previous IVF cycles, embryonic development failure, in the cases of IVF with genetic tests where the patient had no blastocyst development for biopsy, as well as total aneuploidy. The DuoStim protocol was offered with the aim of increasing the number of oocytes and consequently of embryos for transfer or genetic evaluation. Most of these patients had already performed IVF cycles at other clinics before initiating treatment with us, with unfavorable outcomes such as low ovarian response or embryo development failure. Some patients were submitted to IVF in our service, with an unfavorable outcome. The objective of our study was to evaluate the efficacy of the double stimulation protocol over conventional ovarian stimulation in those patients.

## MATERIALS AND METHODS

### Study population

From May 2016 to February 2017, we performed 54 cycles of DuoStim for IVF, and 11 for fertility preservation. Of those 54 IVF DuoStim cases, 13 patients had been previously submitted to IVF with conventional antagonist protocol stimulation in our clinic.

We analyzed the two treatment cycles from each of the 13 patients, comparing the number of oocytes collected, mature oocytes collected, fertilization rates, blastocyst rates, biopsied blastocyst rates and euploidy rates.

The study project was approved by the HUPE Research Ethics Committee.

### Conventional ovarian stimulation antagonist protocol

A baseline transvaginal ultrasound was carried out in the 2^nd^ day of the menstrual cycle, to check ovarian volume, number of antral follicles, presence of residual cysts greater than 15mm and endometrial thickness. Attesting the basal ovarian state, the patient receives the prescription of the medications, which consists of subcutaneous human menopausal gonadotropin (hMG, 75IU) and a subcutaneous injection of recombinant follitropin alpha (FSHr, 225IU). Follicular development was monitored by transvaginal ultrasound starting on day 6 of the cycle and then every two days. Daily administration of GnRH antagonist starts when a follicle reaches 14mm. When at least three follicles reach 16mm in diameter the triggering is carried out with a single subcutaneous injection of recombinant hCG (hCGr, 250mcg), and oocyte retrieval is performed after 35 hours.

### DuoStim protocol

The ovarian double stimulation starts exactly like the conventional protocol, except for the triggering that is carried out with GnRH agonist (triptorelin, 0.2mg). After five days of the first oocyte pick up, the ovarian stimulation restarts with the same protocol. The follow-up of this second stimulation is done as in the first one, with an antagonist beginning with follicles of at least 14mm, and triggering with GnRH agonist from three follicles with at least 16mm.

### IVF laboratory work

After oocyte pick-up and 4 hours of incubation, cumulus and corona radiata cells are removed by hyaluronidase treatment and pipetting, and then the MII oocytes are subjected to ICSI. Fertilization is checked 16 to 18 hours after ICSI and then the presumptive embryos are cultured in groups, up to four embryos, in 25mL of Irvine continuous single culture medium (CSCM; Irvine Scientific, USA), and covered with mineral oil. Culture is performed at 37^o^C in 7,5% carbon dioxide and 5% oxygen tension within a benchtop incubator. Cleavage and embryo score are evaluated on day 3 and the development to the blastocyst stage is evaluated while attesting the viability of the cells or up to day 7. When indicated, the blastocyst biopsy and the chromosome number screening are performed, as follows: on day 4, at morula stage, a 10-20mm hole is opened on the zona pellucida using a diode laser, the embryo goes back to the incubator until the blastocyst expands, when 3 to 7 trophectodermal cells are removed and sent to an outsourced genetics laboratory, in a PCR tube. All embryos, biopsied or not, from the DuoStim protocol are vitrified with the open method (Cryotop or similar).

### Statistical analyses

All statistical analyses were performed in the Excel program, the student *t*-test was used for analysis for interval or reason variables. All numerical variables were expressed as means and standard deviations. The odds ratio (OR) was calculated, and a chi-squared test (χ^2^) was performed for comparison of categorical variables. Fisher's exact test was also performed when necessary. *p* values < 0.05 were considered significant.

## RESULTS

Thirteen patients were analyzed in this study for having performed DuoStim after one cycle of IVF using the antagonist protocol.

The mean age of the study population was 40.9 years, ranging from 37 to 44 years. Five patients were classified as poor responders according to the Bologna criteria (Ferraretti *et al.*, 2011). Of the 13 patients analyzed, one did not perform a pre-implantation genetic test in either treatment, and two had the genetic test indicated only after antagonist cycle failure. The main factor of infertility and the indication of the double stimulation cycle are described in Table 1.

**Table 1:** Treatment characteristics of the study population

Case	Age at conventional (years)	Age at DuoStim (years)	BMI (Kg/m^2^)	Presence of POR	Other infertility factor	PGS conventional	PGS DuoStim	Indication for DuoStim
1	43	44	20	no	Azospermia	yes	yes	Aneuploidy
2	36	38	21	no	Severe endometriosis	no	yes	Embryo accumulation
3	37	37	23	no	Male moderate	yes	yes	Failed development
4	40	40	20	yes	Male terato	no	no	POR
5	41	42	21	no	Male moderate	yes	yes	Embryo accumulation
6	39	39	26	yes	Male terato	no	yes	POR
7	42	43	19	yes	none	yes	yes	POR
8	40	41	22	no	Adenomyosis	yes	yes	Embryo accumulation
9	41	42	23	yes	none	yes	yes	POR
10	38	38	25	no	Severe endometriosis	yes	yes	Failed development
11	43	44	24	yes	Recurrent abortion	yes	yes	POR
12	44	44	23	no	none	yes	yes	Aneuploidy
13	40	41	22	no	none	yes	yes	Aneuploidy
Median (Range)	40 (36- 44)	41 (37- 44)	22 (19-26)					

POR - Poor Ovarian Response. BMI - Body Mass Index. PGS - Pre-implantation genetics screening.

The mean number of oocytes collected was 6.7 in the antagonist cycle and 11.7 in the DuoStim group (*p*=0.007). Of the oocytes collected, the mean number of mature oocytes in the conventional group was 5.3, while in the DuoStim it was 9.23 (*p*=0.01). There was no statistical difference in the rates of fertilization and blastocyst rates, as per shown in Table 2, with a *p* value equal to 0.78 and 0.59, respectively.

**Table 2:** Comparison of laboratorial results of the conventional protocol with DuoStim

	Conventional	DuoStim	*P*-valor	Odds Ratio (CI)
Collected oocytes	6.7 (2-13)*	11.7 (1-28)	0.007	
Mature oocytes	5.3 (2-11)*	9.23 (1-25)	0.010	
% Fertilized	73.6 (51/69)	75.8 (91/120)	0.769	0.90 (0.46-1.78)
% Blastocyst	51.16 (22/43)	46.2 (43/93)	0.593	0.82 (0.40-1.69)

* Median (Range).

Ten patients underwent IVF with genetic testing in both treatment cycles. From the antagonist cycle 20 embryos were biopsied and of these, only two were euploid. In the DuoStim cycles, 32 embryos were biopsied, of which six were euploid. There was no significant difference between the number of embryos biopsied, the number of euploid embryos and euploidy rate, as per described in Table 3.

**Table 3:** Comparison between two catheters for embryo transfer in relation to patient data and IVF cycle outcomes

	Conventional	DuoStim	*p*-valor
N. Biopsied	20	32	0.899
N. euploids	2	6	0.147
% Euploidy	10%	18.7%	0.4634

## DISCUSSION

The purpose of this study was to compare the double stimulation protocol to conventional ovarian stimulation. The evaluated patients had already had unsatisfactory results with the conventional protocol at our clinic, and the opportunity to double stimulate in the same cycle aiming to increase the number of oocytes and embryos was the argument for the new intent to treat.

The number of collected oocytes was one of the factors associated with the positive outcome of assisted reproduction treatment, such as IVF and oocyte cryopreservation. This association between low response and poor embryo quality is not uncommon; it often does not reach the blastocyst stage or arrives in insufficient amounts to achieve an euploid embryo status, leading to cycle and transfers cancellations. This unfavorable situation leads to disappointment, anxiety and frustration, resulting in a high dropout rate (Verberg *et al.*, 2008).

As not only the quality but also the quantity is important, some authors have attempted to estimate an ideal number of oocytes collected, so that patients could achieve the desired pregnancy. This number is even mentioned in studies that try to evaluate the number of oocytes that should be cryopreserved, in cases of oncofertility preservation and for social freezing purposes (Cil *et al.*, 2013). Another important issue in assisted reproduction is the time, which is crucial for patients with rapid loss or decreased fertility. Therefore, a double stimulation to enhance the number of oocytes in the same menstrual cycle is a very attractive strategy.

The opportunity for egg accumulation with double stimulation was demonstrated by Ubaldi *et al*. (2016) and Kuang *et al*. (2014), but both authors performed the statistical analysis between the two stages of stimulation: the follicular and the luteal phases. The main difference of our study was that we evaluated patients who had already been submitted to conventional stimulation, with poor outcomes from that treatment, who had a chance for double stimulation. Our analysis compared the outcome of the two protocols, considering that the double stimulation is the sum of the two phases.

The double stimulation should be considered as a single treatment cycle. Patients who opt for dual stimulation aim at having higher number of oocytes in a shorter period of time, since when performing two independent stimuli the patient must wait for a new menstrual cycle and resolution of the hematic cysts after puncture.

The luteal phase stimulation was believed to affect the oocyte ability to mature and be fertilized. However, as described in other studies, despite the particularities of the DuoStim protocol, there was no difference in fertilization rate and blastocyst rate, confirming the safety in maintaining oocyte quality (Kuang *et al.*, 2014).

In our study, the patients submitted to the DuoStim protocol had a statistically significant increase in the number of oocytes collected, increasing the mean from 5.3 to 9.3 mature oocytes, greater than the minimum described by McAvey *et al*. (2011) to achieve pregnancy. In their study, with 737 women undergoing IVF cycles with less than six MII oocytes, they found a statistically significant decrease in the likelihood of a live birth compared to groups with six or more oocytes. Taking into account that DuoStim was offered to most patients after one year of the last cycle of unsuccessful IVF; it is possible that the number of oocytes obtained would have been higher if DuoStim had been the first option.

Although we have seen an increase in the number of embryos biopsied in the blastocyst stage, we could not reach statistical significance (*p*=0.899), nor did we have significant fertilization and blastocyst rates between the two protocols. We believe that it happened due to the small number of cases analyzed. Since a prospective study with these characteristics would not be ethical, for not offering what we believe to be the best for the patient in the group that would be randomized to the traditional protocol.

## CONCLUSION

Double stimulation favors patients who would need more than one stimulus to produce an adequate amount of oocytes. The greatest benefit of this protocol is the accumulation of oocytes in a single cycle of stimulation, minimizing the time in which it will be performed. In addition, it allows the production of a larger number of embryos, which can then be genetically evaluated, thus favoring the final clinical result.
